# A network analysis of the long-term quality of life and mental distress of COVID-19 survivors 1 year after hospital discharge

**DOI:** 10.3389/fpubh.2023.1223429

**Published:** 2023-07-28

**Authors:** Pu Peng, Yaqi Wang, Zhuqing Li, Yanan Zhou, Ji Wang, Miao Qu, Tieqiao Liu

**Affiliations:** ^1^Department of Psychiatry, National Clinical Research Center for Mental Disorders, and National Center for Mental Disorders, The Second Xiangya Hospital of Central South University, Changsha, Hunan, China; ^2^College of Basic Medical Sciences, Zhejiang Chinese Medical University, Hangzhou, China; ^3^National Institute of Traditional Chinese Medicine Constitution and Preventive Treatment of Diseases, Beijing University of Chinese Medicine, Beijing, China; ^4^Department of Psychiatry, Hunan Brain Hospital (Hunan Second People’s Hospital), Changsha, China; ^5^Department of Neurology, Xuanwu Hospital of Capital Medical University, Beijing, China

**Keywords:** network analysis, COVID-19, fatigue, quality of life, depression, mental distress

## Abstract

**Objectives:**

COVID-19 survivors suffer from persistent mental distress and impaired quality of life (QOL) after recovery from the infection. However, the symptom-symptom interaction between these psychological variables remained unexplored. The present study aimed to determine the symptom network of mental distress (depression, anxiety, sleep disturbance, fatigue, and post-traumatic stress disorder) and their association with QOL among 535 COVID-19 survivors 1 year after hospital discharge.

**Methods:**

9-item Patient Health Questionnaire, 7-item Generalized Anxiety Disorder Scale, Chalder fatigue scale, Impact of Event Scale-Revised, Pittsburgh Sleep Quality Index, and 36-Item Short-Form Health Survey were applied to measure depression, anxiety, fatigue, PTSD, sleep disturbances, and QOL, respectively. Two networks were estimated using Gaussian graphical model. Network 1 consisted of mental symptoms to determine the central and bridge symptoms. Network 2 additionally included QOL to determine which mental symptoms were mostly related to QOL.

**Results:**

60% of the COVID-19 survivors experienced mental distress 1 year after hospital discharge. Uncontrollable and excessive worry, psychomotor symptoms, intrusion, and daytime dysfunction were the most central symptoms. Daytime dysfunction and fatigue (especially mental fatigue and loss of energy) served as the bridge symptoms across the mental distress network and exhibited the most substantial association with QOL.

**Conclusion:**

Our study demonstrated several key symptoms that played a vital role in mental distress and QOL among COVID-19 survivors. Prompt screening and targeted interventions for these symptoms might hold great promise in preventing mental distress and improving QOL in COVID-19 survivors.

## 1. Introduction

Approximately 30% of COVID-19 survivors experience mental health sequelae of COVID-19 infection, such as fatigue, depression, sleep disturbance, and anxiety (1–3). Unfortunately, these symptoms could be persistent even long after the recovery from the primary infection. According to a meta-analysis, a large proportion of COVID-19 survivors still experience fatigue (28%), depression (23%), and anxiety (22%) 1 year after infection (4). Prolonged mental distress takes a considerable toll on the quality of life (QOL) of COVID-19 survivors (5, 6). China was the first country to be affected by the COVID-19 pandemic. Several studies have highlighted the long-term mental health sequelae among Chinese COVID-19 survivors (7–9). For example, Huang et al. demonstrated that approximately one-fourth of the COVID-19 survivors experienced anxiety and depressive symptoms 1 year after their infection (9). Another recent study reported that 27 and 36% of the Chinese COVID-19 survivors suffered from long-term depression and anxiety, which was closely associated with stigma (8). Compared to non-infection individuals, COVID-19 survivors were at a much higher risk for mental distress and low QOL (7). Hence, early identification and intervention for mental distress in COVID-19 survivors are vital.

Despite the high interest in long-term post-COVID-19 mental distress and its association with QOL, very few studies have elucidated their interrelationship at a symptom level. Most of the previous studies used the total scores of measurement tools as an index of mental distress (e.g., summing up all items in a depression scale and interpreting the summed scores as the severity of depression). However, the use of the total scores assumes that all symptoms had equal importance, which fails to point out the potential difference and interaction between mental symptoms (10). Understanding whether and how mental symptoms interact and reinforce each other, and how they relate to impaired QOL, may provide new insights into preventing and treating mental distress and improving QOL in COVID-19 survivors.

Network analysis is a promising tool for understanding mental distress at a symptom level (11, 12). It assumes mental distress is composed of interacting symptoms. Network analysis allows us to quantify the importance of each symptom by identifying the “central symptoms,” which are most associated with and impose the most substantial impact on other symptoms within the network (13). It also provides new insights into the establishment of comorbidity. By modeling symptoms of different mental distress into the network, network analysis can determine the bridge symptoms that connected different constructs. The central and bridge symptoms are important targets for intervention as they play a vital role in triggering and maintaining the network of distress (14, 15).

To date, emerging studies have documented the network structure of mental distress (mainly depression and anxiety) in different populations such as the general population (16), adolescents (17), college students (18), and healthcare workers (19, 20) during the COVID-19 pandemic. However, to our knowledge, only two studies assessed the network of mental distress in COVID-19 patients (21, 22). Both studies recruited patients with recent infections (patient’s 1-month after discharge or patients in the Fangcang shelter hospitals). The network of long-term post-COVID-19 mental distress among COVID-19 survivors remains largely unexplored.

Therefore, we conducted the present study to map the network of several common mental distress [depression, anxiety, fatigue, sleep disturbances, and posttraumatic disorder (PTSD)] and QOL among a large sample of COVID-19 survivors 1 year after their hospital discharge. We had two main aims: (1) to determine the central and bridge symptoms within the mental distress network among COVID-19 survivors; (2) to determine which symptoms were most closely related to impaired QOL.

## 2. Methods

### 2.1. Study procedure and participants

COVID-19 survivors were recruited from April to May 2021. The inclusion criteria were as follows: (1) diagnosed with COVID-19 according to the Chinese standard (23); (2) discharged from Huanggang Central Hospital from April to May 2020; and (3) willing and able to participate in the online survey and to provide informed consent. Huanggang Central Hospital is a tertiary hospital, serving as the largest healthcare facility in Huanggang, Hubei. It had approximately 1,500 beds and served as the primary medical center for local COVID-19 patients during the pandemic. From April to May 2020 when the participants were discharged, the COVID-19 pandemic in Hubei entered its temporary remission period. There was no striction on the age and sex. Participants were recruited with the assistance of their primary doctors during hospitalization, who were responsible for introducing the study purpose, distributing the questionnaire, providing instructions on how to complete it, and addressing any participants’ inquiries. Participation was voluntary, and participants could withdraw from the survey at any time. Informed consent was obtained from all participants, and for those under 18 years old, consent was also obtained from their legal guardians prior to the start of the study. The study protocol was reviewed and approved by the Institutional Review Board of the Ethics Committee of Beijing University of Traditional Chinese Medicine (2020BZHYLL0111).

### 2.2. Measurements

#### 2.2.1. Basic information

We collected the following basic information through a self-designed questionnaire: age, sex, body mass index (BMI), family member, education levels, income levels, employed status, smoking status, alcohol use status, history of physical illness, and severity of COVID-19.

#### 2.2.2. Mental distress

Depression and anxiety symptoms were measured through the 9-item Patient Health Questionnaire (PHQ-9) and 7-item Generalized Anxiety Disorder Scale (GAD-7). Both scales applied a four-Likert scale. Participants rated the frequency of their symptoms within the last 2 weeks, ranging from 0 (Not at all) to 3 (Almost every day). The total scores of PHQ-9 and GAD-7 ranged from 0 to 27 and 0 to 21, respectively. Higher scores indicated more severe symptoms. PHQ9 and GAD7 were highly validated and widely used measurement tools in the Chinese population (18). Following a previous study (17), a cutoff point of 10 for PHQ-9 and GAD-7 was used to detect the presence of depressive and anxiety symptoms.

We used Pittsburgh Sleep Quality Index (PSQI) to assess sleep disturbance (24). PSQI was composed of 21 items, grouped into seven factors: subjective sleep quality, sleep latency, sleep duration, low sleep efficacy, sleep disturbances, sleep medication, and daytime dysfunction. The scores of each factor ranged from 0 to 3. PSQI scores above 5 indicated severe sleep disturbance.

The impact of event scale-revised (IES-R) was applied to evaluate the post-traumatic stress disorder (PTSD) symptoms (25). IES-R consisted of 22 items and three subscales, including intrusion, avoidance, and hyperarousal. IES-R employed a five-Likert scale, ranging from 0 (Not at all) and 4 (Always). Participants with IES-R scores above 32 were considered as having clinically relevant PTSD symptoms.

Fatigue was assessed through the 11-item Chalder fatigue scale (CFQ) (26). Responders answered each CFQ item with 0 (absence) and 1 (presence) to indicate whether they experienced fatigue symptoms. Following a previous study in the Chinese population (27), CFQ was divided into three factors including physical fatigue (CFQitems 1–3), loss of energy (CFQitems 4–7), and mental fatigue (CFQitems 8–11). A cutoff point of 4 was employed to detect severe fatigue. Those who had at least one type of mental distress (depression, anxiety, sleep disturbance, PTSD, and fatigue) were categorized into the mental distress group.

#### 2.2.3. Quality of life

We used the 36-Item Short-Form Health Survey (SF-36) to measure the quality of life (QOL) of COVID-19 survivors, which was widely used in the COVID-19 survivors (28). It measured nine dimensions of QOL, including physical functioning (PF), role-physical (RF), bodily pain (BP), general health (GH), vitality (VT), social functioning (SF), role-emotional (RE), mental health (MH), and reported health transition (HT). Higher scores indicated better QOL.

### 2.3. Statistical analysis

We described the continuous and categorical data as median (quartiles) and frequency (percentage), respectively. Basic information and QOL were compared between COVID-19 survivors with and without mental distress using Chi-square tests and Wilcoxon rank sum test as appropriate. Bonferroni tests were applied for multiple comparisons (*p*’ = 0.05/17 = 0.0029). All statistical analysis was performed on R (ver. 4.2.2). The tests were two-tailed, with *p* < 0.05 indicating statistical significance.

### 2.4. Network analysis

We conducted two networks. Network1 was composed of depressive, anxiety, sleep disturbance, PTSD, and fatigue symptoms. In the second network, we added the QOL variables (subscales of SF-36) to further determine which symptom had the most substantial impact on QOL.

#### 2.4.1. Network estimation

Two items in PHQ9 were excluded from the network analysis as they overlapped with PSQI and CFQ: PHQ3 (Sleep trouble) and PHQ4 (Fatigue). In addition, we excluded the “sleep medication” factor in PSQI as previous studies suggested that it measured the indirect sleeping behavior (29). “goldbricker” function in Rpackage “networktools” was employed to determine the redundancy of PHQ-9 items, GAD-7 items, IES-R items, CFQ factors, and PSQI factors. As a result, we found there were substantial overlaps in IES-R items. Therefore, we calculated the mean score of the three subscales of IES-R and entered them into the network. Finally, network 1 was modeled with seven items of PHQ-9, seven items of GAD-7, six factors of PSQI, three factors of CFQ, and three factors of PTSD. Network 2 was modeled with mental distress and nine subdomains in SF-36.

The Rpackage “bootnet” and “qgraph” was used to estimate and visualize the network. Following previous studies (14, 19), the Gaussian graphical model using extended Bayesian Information Criterion (EBIC) model selection was employed. The cormethod was set to “spearman” as the data were ordinal. In the network, each “node” represented a symptom, and the “edge” indicated the unique association between the two nodes. The presence of an edge linking two nodes suggested that the two symptoms remained independently related after full adjustment for other nodes within the network (30). The thickness and the color of the edges indicated the direction and strength of association. Blue edges indicated positive associations while red edges suggested negative associations. Thicker edges suggested more substantial associations.

#### 2.4.2. Central and bridge symptoms in the network of mental distress

We calculated node “Expected Influence” (EI) by summing up the weights of all edges directly related to that node, including both positive and negative associations (31). Symptoms with high EI were considered central symptoms, which imposed higher impacts on other symptoms within the network. Bridge expected influence (BEI) was also calculated in a similar way with EI, which only included edges connecting a node to nodes in other constructs (e.g., the BEI of PHQ1 was determined by summing up edges connected it to nodes in fatigue, GAD-7, PSQI, and PTSD). BEI allowed us to examine which symptoms could serve as bridges across different constructs. Nodes with high BEI were considered as the bridge symptoms, which could drive the comorbid. Following a previous study (32), a 90th percentile cutoff of BEI was applied to determine the bridge symptoms across the network. We also calculated the predictability of each item of Network 1 using the Rpackage “MGM.” Similar to *R*^2^ in the regression model, predictability described how the variance of the node could be explained by other nodes within the network. Nodes with high predictability might be more easily controlled by targeting their related nodes.

#### 2.4.3. Network stability and accuracy

We employed the case-dropping procedure to evaluate the stability of the network. The correlation stability coefficient (CS-C) for EI and BEI was calculated, with CS-C above 0.5 suggesting nice network stability. Nonparametric bootstrapping with 1,000 bootstrap samples were applied to determine the accuracy of the estimated edges within the network.

#### 2.4.4. Network of mental distress and quality of life

In the end, we added SF-36 into the network. Network 2 was composed of 35 nodes (26 in the mental distress community and 9 in the QOL community). We calculated the BEI to determine the bridge node across the two communities. We assumed that most of the associations between mental symptoms and QOL were negative. Symptoms with a more negative BEI value were considered as imposing more substantial impairments on quality of life. We also conducted a *post hoc* analysis by including the demographic characteristics into the network to further illustrate the association of mental symptoms and QOL in COVID-19 survivors. Variables that exhibited significant associations with mental distress in the univariate analysis were chosen.

## 3. Results

### 3.1. Descriptive analysis

Among 566 survivors who were screened, 535 participated in the survey, yielding a response rate of 95%. Table 1 described the basic information, mental health status, and QOL of the participants. The incidence of depression, anxiety, PTSD, sleep disturbance, and fatigue was 21% (*n* = 112), 16% (*n* = 84), 24% (*n* = 130), 47% (*n* = 252), and 37% (*n* = 200), respectively. 58% (*n* = 313) of the participants experienced at least one mental distress. There was a significant difference in age, BMI, monthly income, smoking status, alcohol consumption, and history of chronic physical illness between COVID-19 survivors with and without mental distress, which remained significant after Bonferroni’s tests. Participants with mental distress had much worse QOL than those without (all *p* < 0.001).

**Table 1:** Sample characteristics of participants with and without mental problems (depression, anxiety, fatigue, sleep disturbance, and PTSD).

Variable	Overall, *N* = 535^1^	Without mental problems, *N* = 222^1^	With mental problems, *N* = 313^1^	*p* value^2^	Adjusted *p*’ value^3^
Gender				0.5	>0.05
Female	319 (60%)	129 (58%)	190 (61%)		
Male	216 (40%)	93 (42%)	123 (39%)		
Age, year				<0.001	<0.05
10–39	120 (22%)	58 (26%)	62 (20%)		
40–59	281 (53%)	130 (59%)	151 (48%)		
60–99	134 (25%)	34 (15%)	100 (32%)		
BMI, kg/m^2^				<0.001	<0.05
≥23	221 (41%)	73 (33%)	148 (47%)		
<23	314 (59%)	149 (67%)	165 (53%)		
Education level				0.005	>0.05
Below college	382 (71%)	144 (65%)	238 (76%)		
College or above	153 (29%)	78 (35%)	75 (24%)		
Family member				0.4	>0.05
>3	215 (40%)	94 (42%)	121 (39%)		
≤3	320 (60%)	128 (58%)	192 (61%)		
Income (month/yuan)				<0.001	<0.05
0–2,999	346 (65%)	113 (51%)	233 (74%)		
≥3,000	189 (35%)	109 (49%)	80 (26%)		
Current employment status				<0.001	<0.05
Employed	294 (55%)	146 (66%)	148 (47%)		
Unemployed	126 (24%)	48 (22%)	78 (25%)		
Retirement	115 (21%)	28 (13%)	87 (28%)		
Current smoking				<0.001	<0.05
Smoker	65 (12%)	14 (6.3%)	51 (16%)		
Non-smoker	470 (88%)	208 (94%)	262 (84%)		
Current alcohol use				0.002	<0.05
Drinker	45 (8.4%)	9 (4.1%)	36 (12%)		
Non-drinker	490 (92%)	213 (96%)	277 (88%)		
History of chronic illness				<0.001	<0.05
With	188 (35%)	35 (16%)	153 (49%)		
Without	347 (65%)	187 (84%)	160 (51%)		
Severity of COVID-19				0.4	>0.05
Mild	84 (16%)	39 (18%)	45 (14%)		
Moderate	358 (67%)	147 (66%)	211 (67%)		
Severe	92 (17%)	35 (16%)	57 (18%)		
Critical	1 (0.2%)	1 (0.5%)	0 (0%)		
PHQ9 scores	4 (0, 9)	0 (0, 1.8)	8 (4, 14)	<0.001	<0.05
GAD7 scores	1 (0, 7)	0 (0, 0)	5 (1, 11)	<0.001	<0.05
CFQ scores	2 (1, 6)	1 (0, 1)	5 (1, 8)	<0.001	<0.05
PSQI scores	5 (3, 8)	3 (2, 4)	8 (6, 11)	<0.001	<0.05
IESR scores	21 (10, 32)	13 (5, 21)	27 (18, 42)	<0.001	<0.05
SF36 scores	687 (581, 763)	770 (720, 807)	612 (498, 687)	<0.001	<0.05

1 *n* (%); Median (IQR).

2 Pearson’s Chi-squared test; Wilcoxon rank sum test.

3 Adjust for Bonferroni’s tests.

BMI, body mass index; PHQ9, patient health questionnaire; GAD-7, generalized anxiety disorder scale; CFQ, Chalder fatigue scale; PSQI, Pittsburgh sleep quality index; IESR, impact of event scale-revised; SF-36, 36-item short-form health survey.

### 3.2. The network structure of mental distress

**Figure 1:**
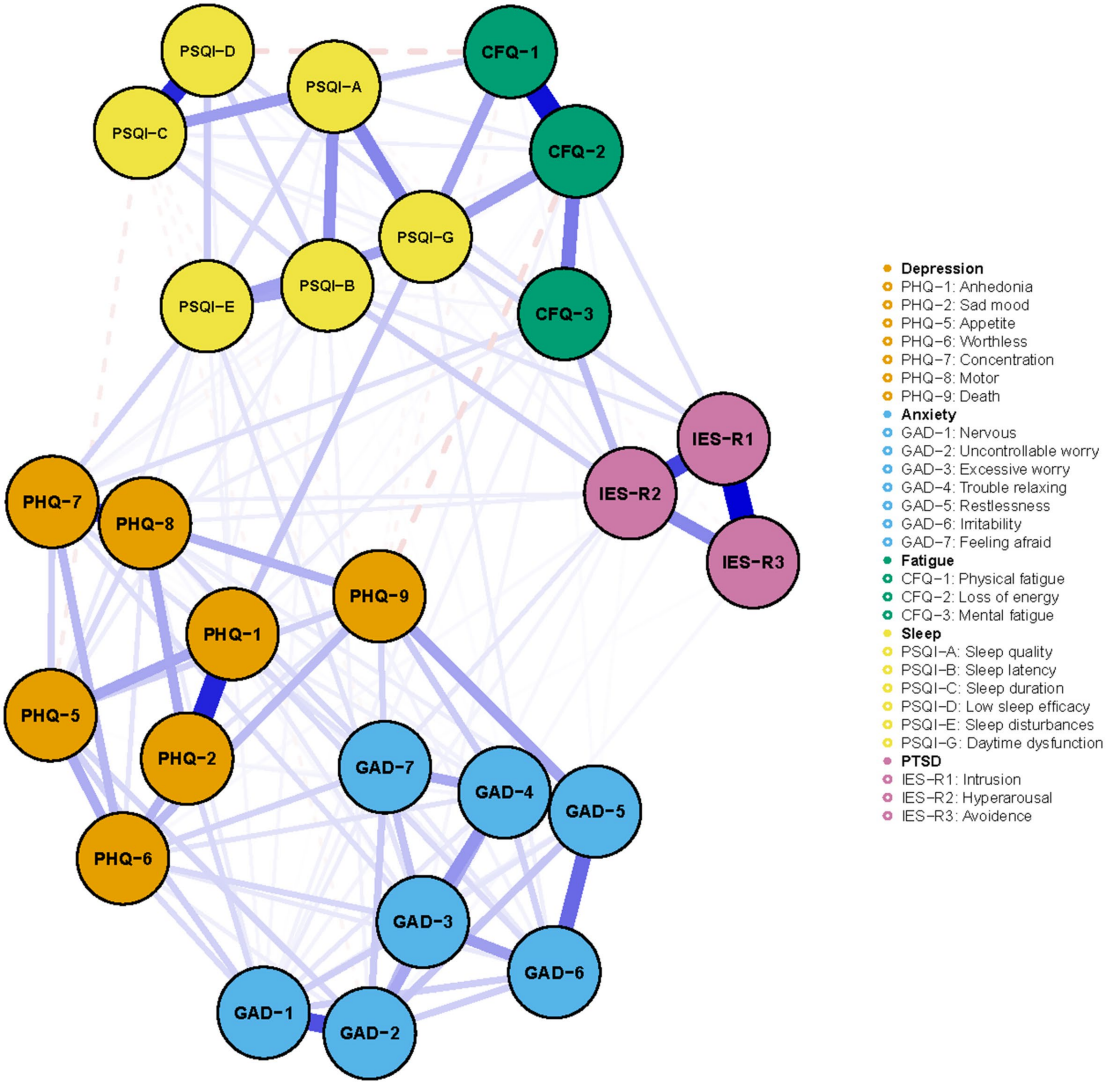
The network of mental distress among COVID-19 survivors. The network structure of mental distress. Orange, blue, green, yellow, and purple nodes represented depression, anxiety, fatigue, sleep disturbances, and PTSD symptoms, respectively. The blue edges suggested positive association, while the red, dash edges indicated negative association. Thicker edges implied stronger association.

The detailed contents of the mental symptoms included in the network was displayed in Table 2. All PHQ-9, GAD-7, PSQI, CFQ, and IES-R items/factors were not normally distributed. Figure 1 illustrated the network of depression, anxiety, sleep disturbance, fatigue, and PTSD symptoms among COVID-19 survivors. The network was composed of 26 nodes, with a density of 0.46 (152/325 edges). The mean weight of the estimated edges was 0.037. All the strongest edges were within the respective distress. The most substantial edges were IESR1 (Intrusion)—IESR3 (Avoidance), followed by CFQ1 (Physical fatigue)—CFQ2 (Loss of energy), PHQ1 (Anhedonia)-PHQ2 (Sad mood), PSQI-D (Low sleep efficacy)-PSQI-C (Sleep duration), IESR2 (Hyperarousal)-IESR (Intrusion), PHQ7 (Concentration)-PHQ8 (Psychomotor symptoms), and GAD1 (Nervous)-GAD2 (Uncontrollable worry). These edges were statistically stronger than other edges within the network according to the nonparametric bootstrapping procedure. The edges PSQI-G (Daytime dysfunction)-CFQ2 (Loss of energy), PSQI-G (Daytime dysfunction)-CFQ1 (Physical fatigue), GAD5 (Restlessness)-PHQ9 (Death), IESR2 (Hyperarousal)-CFQ3 (Mental fatigue), and PSQI-G (Daytime dysfunction)-PHQ1 (Anhedonia) were the strongest transdiagnostic edges across the network. Supplementary Table S1 described the correlation matrix of the items in Network 1.

**Table 2:** Descriptive statistics of the items in the network of mental distress.

Items	Item content	Mean	SD	Skewness	Expected influence	Predictability
PHQ1	Anhedonia	0.76	0.90	0.98	0.52	0.78
PHQ2	Sad mood	0.70	0.88	1.08	0.07	0.79
PHQ5	Appetite	0.61	0.82	1.13	−0.74	0.73
PHQ6	Worthless	0.58	0.81	1.21	0.61	0.81
PHQ7	Concentration	0.64	0.87	1.10	0.31	0.76
PHQ8	Motor	0.55	0.81	1.27	1.27	0.82
PHQ9	Death	0.40	0.77	1.86	−1.46	0.76
GAD1	Nervous	0.63	0.81	1.10	0.73	0.86
GAD2	Uncontrollable worry	0.59	0.81	1.16	1.28	0.88
GAD3	Excessive worry	0.60	0.84	1.24	1.06	0.84
GAD4	Trouble relaxing	0.57	0.83	1.29	0.28	0.84
GAD5	Restlessness	0.51	0.80	1.43	0.84	0.86
GAD6	Irritability	0.60	0.83	1.20	0.61	0.84
GAD7	Feeling afraid	0.47	0.79	1.59	−0.18	0.82
CFQ1	Physical fatigue	1.06	1.21	0.58	−1.32	0.59
CFQ2	Loss of energy	1.23	1.58	0.81	0.86	0.68
CFQ3	Mental fatigue	1.13	1.07	1.01	−1.65	0.52
PSQI-A	Sleep quality	1.06	0.72	0.43	0.00	0.55
PSQI-B	Sleep latency	1.27	0.84	0.45	−0.80	0.47
PSQI-C	Sleep duration	0.83	0.79	1.00	−1.27	0.51
PSQI-D	Low sleep efficacy	0.85	1.08	0.91	−1.85	0.45
PSQI-E	Sleep disturbances	1.12	0.82	0.54	−0.80	0.52
PSQI-G	Daytime dysfunction	0.87	0.90	0.83	0.87	0.64
IESR1	Intrusion	1.02	0.80	0.46	1.12	0.76
IESR2	Hyperarousal	0.84	0.83	0.71	0.78	0.74
IESR3	Avoidance	1.01	0.87	0.54	−1.14	0.67

PHQ9, patient health questionnaire; GAD-7, generalized anxiety disorder scale; CFQ, Chalder fatigue scale; PSQI, Pittsburgh sleep quality index; IESR, impact of event scale-revised.

**Figure 2:**
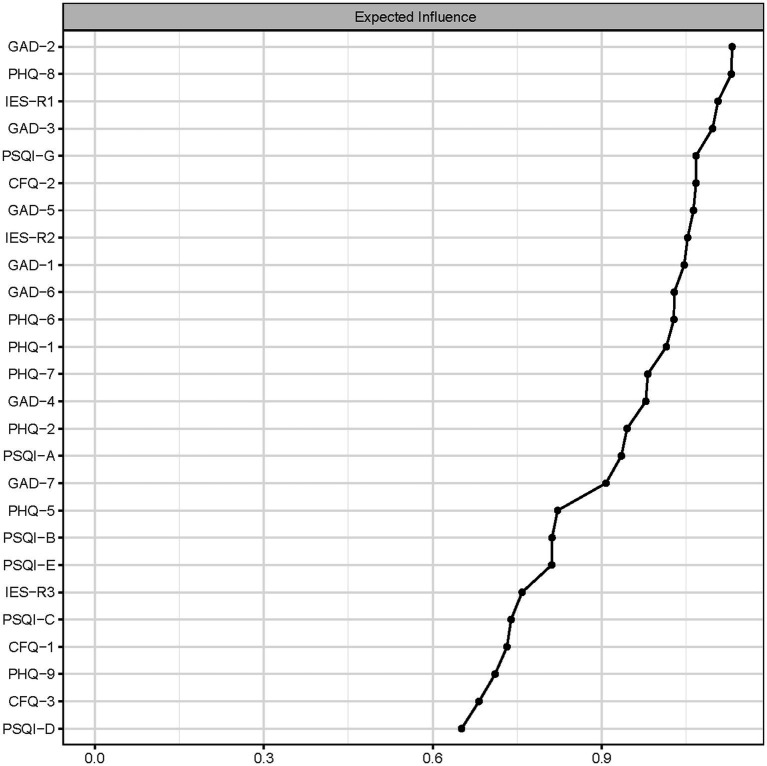
The centrality plot of network. Nodes with higher expected influence have stronger impact in other nodes within the network.

**Figure 3:**
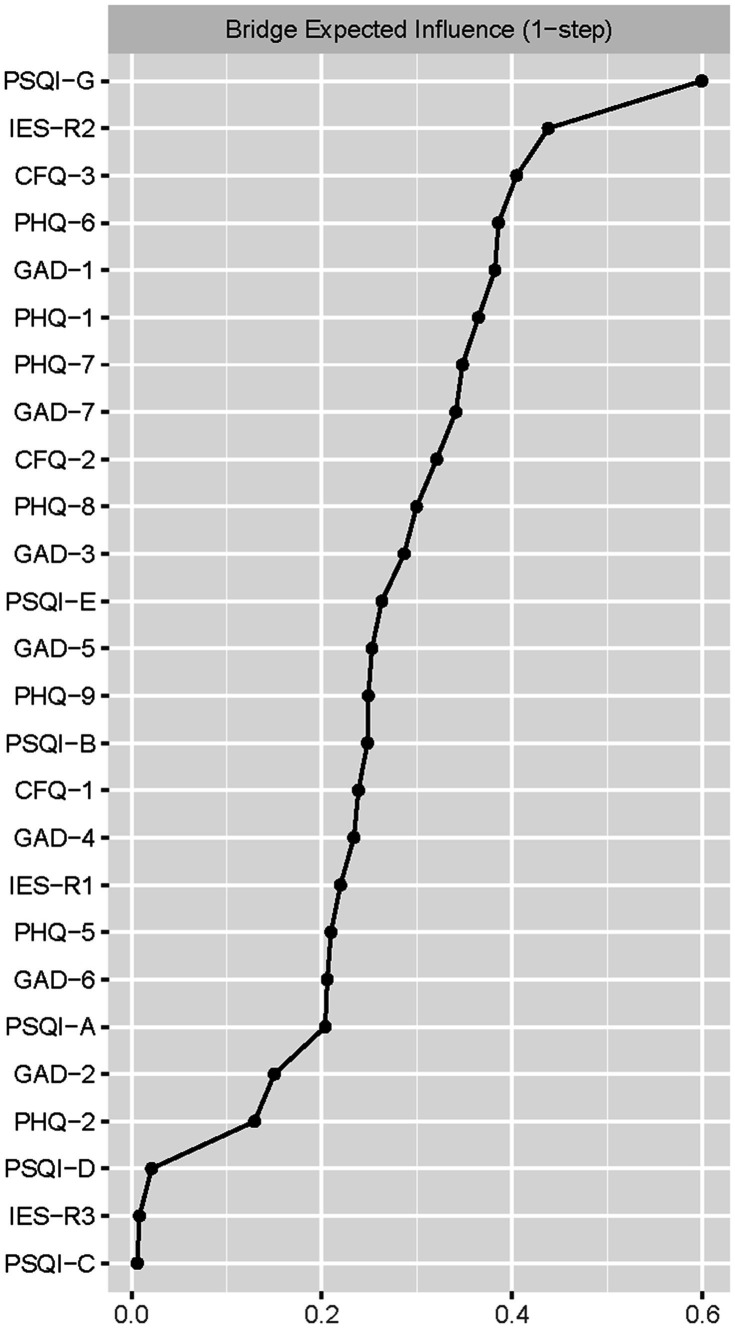
The bridge expected influence plot. Nodes with higher bridge expected influence are recognized as bridge symptoms that drove the comorbid mental distress.

Figure 2 presented the centrality plot of Network 1. GAD2 (Uncontrollable worry), PHQ8 (Psychomotor symptoms), IES-R1 (intrusion), GAD3 (Excessive worry), and PSQI-G (Daytime dysfunction) had the highest EI and were recognized as central symptoms. However, their EI did not statistically differ from each other (Supplementary Figure S1). According to the BEI (Figure 3), PSQI-G (Daytime dysfunction), IESR2 (Hyperarousal), and CFQ3 (Mental fatigue) were the bridge symptoms across the network. The mean predictability of the nodes was 0.71 (Table 2), suggesting 71% of the variance of the nodes within the network could be predicted by their neighboring nodes. PSQI-D (Low sleep efficacy) held the lowest predictability (0.45), while GAD2 (Uncontrollable worry) had the highest (0.88).

The network has nice stability and accuracy. The CS-C of the network EI was 0.75, suggesting that the network remained stable after 75% of the raw data were dropped (Supplementary Figure S2). The accuracy of the edges estimated according to the nonparametric bootstrapping was presented in Supplementary Figure S3. The 95% CIs were narrow and non-overlapping, indicating a nice edge accuracy.

### 3.3. The network structure of mental distress and quality of life

**Figure 4:**
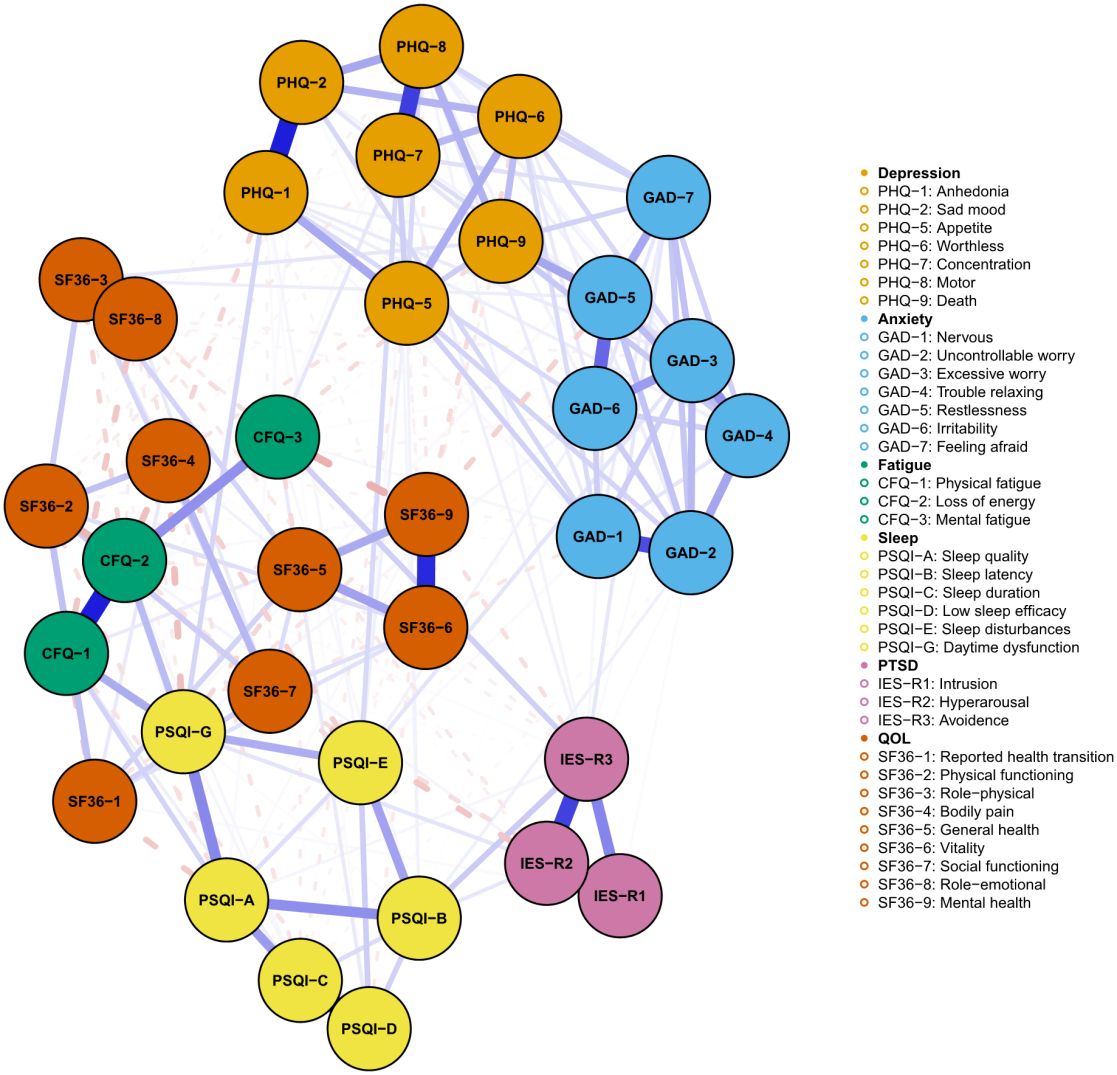
Network of mental distress and QOL among COVID-19 survivors. The network structure of mental distress and QOL. Orange, blue, green, yellow, purple, and red nodes represented depression, anxiety, fatigue, sleep disturbances, PTSD symptoms, and QOL, respectively. The blue edges suggested positive association, while the red, dash edges indicated negative association. Thicker edges implied stronger association.

**Figure 5:**
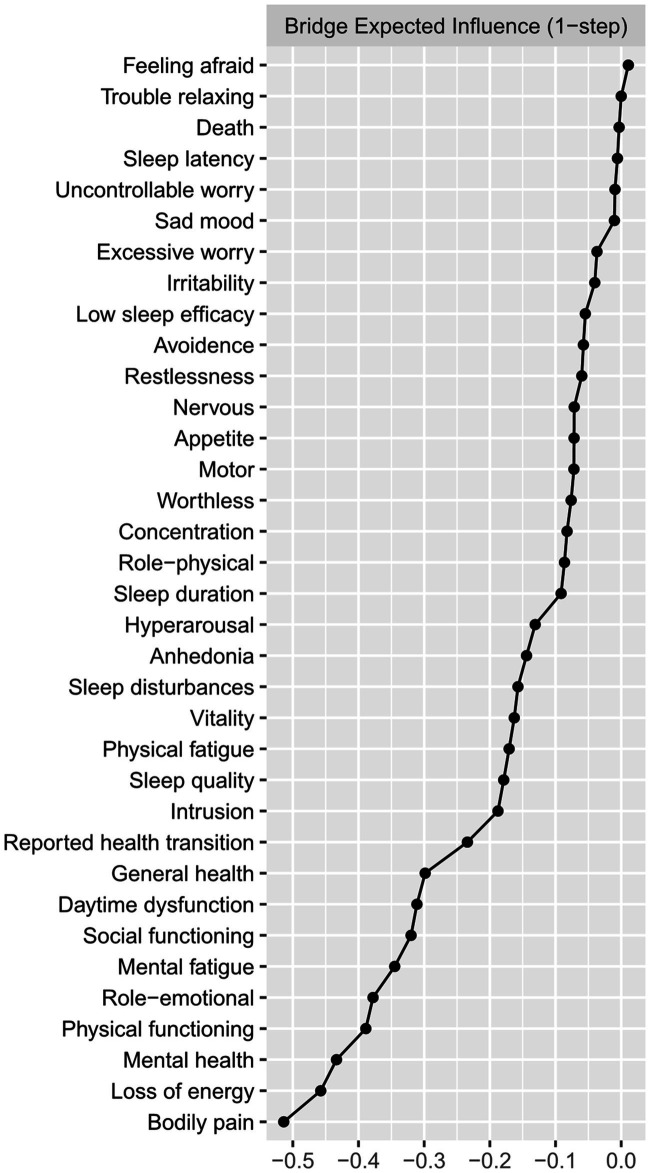
The bridge plot of network of mental distress and QOL. Nodes with more negative BEI value were considered to have more impacts on QOL.

Figures 4, 5 presented the network of mental distress and QOL. The network was composed of 35 nodes, which had a density of 0.39 (231/595) and a mean weight of 0.018. The most substantial edges linking mental distress and impaired QOL were MH-CFQ3 (Mental fatigue), followed by PF-CFQ2 (Loss of energy), BP-PSQI-G (Daytime dysfunction), BP-CFQ2 (Loss of energy), and SF-IESR2 (Hyperarousal). Supplementary Table S2 provided a detailed correlation between QOL and mental symptoms. CFQ2 (Loss of energy), CFQ3 (Mental fatigue), and PSQI-G (Daytime dysfunction) had the most negative BEI among the mental distress communities, suggesting that these symptoms were closely related to worse QOL. Similarly, bodily pain, mental health, and physical function exhibited the most negative BEI in QOL communities, which suggested that they were the most affected QOL subdomain in COVID-19 survivors. After adjusting demographic information that was closely associated with mental distress (age, BMI, monthly income, smoking status, alcohol consumption, and history of chronic physical illness) in the network, CFQ2 (Loss of energy), CFQ3 (Mental fatigue), and PSQI-G (Daytime dysfunction) remained to be the bridge symptoms with highest negative BEI that linked QOL and mental distress in COVID-19 survivors (Supplementary Figures S4, S5). CFQ2 (Loss of energy) exhibited substantial association with five out of nine subdomains of QOL (PF, RP, BP, GH, and SF). Furthermore, PSQI-G (Daytime dysfunction) and CFQ3 (Mental fatigue) predicted significant impairments in four (HT, RP, BP, and GH) and three (RP, GH, and MH) subdomains of QOL, respectively.

## 4. Discussion

To our knowledge, our study provides the first network-based analysis of the long-term mental health sequelae of COVID-19 infection and its association with impaired QOL. Our major findings included: (1) approximately 60% of the COVID-19 survivors suffered from at least one type of mental distress (depression, anxiety, fatigue, PTSD, and sleep disturbances) 1 year after hospital discharge. COVID-19 survivors with mental distress had much worse QOL than those without; (2) Uncontrollable and excessive worry, psychomotor symptoms, intrusion, and daytime dysfunction were the most central symptoms of the mental distress network; and (3) Daytime dysfunction and fatigue (especially mental fatigue and loss of energy) served as bridge symptoms linking different mental distresses. They were also the most predominant symptoms negatively associated with impaired QOL among COVID-19 survivors.

Our study suggested that the most central symptom within the network was uncontrollable worry, which aligned with previous network analyses of depression and anxiety symptoms among other populations during the COVID-19 pandemic (17, 33). Similarly, a recent study suggested that uncontrollable worry was one of the core symptoms in the network of depression-anxiety-stress among patients with recent COVID-19 infection (21). A possible explanation for the high centrality of uncontrollable worry in COVID-19 patients might be the COVID-19-related fear, such as reinfection and sequelae. During the data collection periods, highly contagious variants of COVID-19 and various COVID-19 sequelae were reported, which might trigger the anxiety of the participants (34).

Our study demonstrated a high incidence of depression (21%), anxiety (16%), PTSD (24%), sleep disturbance (47%), and fatigue (37%) among COVID-19 survivors. The incidence of these long-term mental distress was fairly close to previous reports in both Chinese and global populations of COVID-19 survivors (4, 7–9). The prevalence of any mental distress was 58%, which was much higher than that in the Chinese general population (35).

Our study demonstrated that daytime dysfunction played an important role in mental distress and impaired QOL among COVID-19 survivors. It served as both central and bridge symptoms within the network of mental distress, exhibiting tight associations with anhedonia, physical fatigue, loss of energy, low sleep quality, and sleep disturbances. In contrast, other nocturnal symptoms such as sleep latency and sleep efficacy held lower EI and BEI in the network of mental distress among COVID-19 survivors. This finding indicated that the sleep-related symptoms might mainly manifest as daytime disturbances such as sleepiness in COVID-19 survivors. A comparative study in sleep disturbances between COVID-19 patients and non-COVID-19 population supported our results, which found significant differences in daytime dysfunction rather than nocturnal symptoms including sleep latency, sleep duration, and sleep efficacy between the two groups (36). Further large-scale studies in COVID-19 survivors were needed to verify our findings. In addition, daytime dysfunction and other sleep-related symptoms such as low sleep quality displayed substantial negative associations with QOL, which was in line with the study of Bai et al. (37) among Macau residents during the COVID-19 pandemic. Hence, targeted interventions aimed at daytime dysfunction might be particularly useful in both revealing mental distress and improving impaired QOL among COVID-19 survivors.

Fatigue was one of the most common post-COVID-19 mental symptoms in COVID-19 survivors (4). Our study suggested that fatigue, especially loss of energy and mental fatigue, were predominantly associated with impaired QOL among COVID-19 survivors, which replicated previous findings in the general population during the pandemic (38, 39). The multidimensional nature of fatigue and the overlap between fatigue and symptoms of depression made it hard to evaluate the relationship between fatigue and other mental distress (40). Our network analysis suggested that different dimensions of fatigue exhibited varying levels with other mental symptoms, which indicated that targeted interventions for different types of fatigue were needed. Physical fatigue was mainly associated with daytime dysfunction and subjective sleep quality. Further, the relationship between physical fatigue and mental fatigue was rather weak, suggesting that they might be distinct constructs. The relationship between physical fatigue and depressive, anxiety, and PTSD symptoms was absent or weak. Loss of energy was associated with most of the sleep problems and intrusion. At last, we observed a strong association of mental fatigue with hyperarousal and worthless feelings. Taken together, targeting sleep problems, especially sleep dysfunction and sleep quality might help reduce physical fatigue and loss of energy, while patients with mental fatigue might benefit from intervention against PTSD symptoms. However, our conjecture requires further investigations to verify.

Our study has several implications. First, our study suggested the high prevalence of mental distress among COVID-19 survivors 1 year after their hospital discharge, which called for continuous monitoring and intervention in this population. Second, our study revealed the relationship between depression, anxiety, sleep disturbances, fatigue, PTSD, and QOL at a symptom level. Several central symptoms, such as uncontrollable and psychomotor symptoms, were identified and should receive higher priority for screening and intervention. Notably, daytime dysfunction and fatigue emerged as the bridge symptoms linking different mental problems and had the most profound negative impacts on QOL. Hence, targeting them might hold promise in reducing mental distress and improving QOL among COVID-19 survivors. For example, a recent network intervention analysis suggested that cognitive therapy was superior in revealing daytime dysfunction than behavior therapy (41), which was a potential treatment option among COVID-19 survivors. Previous studies have suggested the efficacy of exercise on fatigue (42, 43), which might also hold promise in relieving mental distress and promoting QOL.

Several limitations should be acknowledged. First, the cross-sectional study design prohibited us from drawing causal relationships. Second, this study was conducted at a single center in China. All participants were previously hospitalized patients during the first wave of the pandemic. It remains uncertain how our findings could be applied to COVID-19 survivors with different virus strains and cultural background. Future studies could benefit from incorporating more recent and cross-cultural data to capture potential changes due to the virus’s evolution over time and to determine the potential impact of cultural background on mental health and QOL among COVID-19 survivors. Third, we did not collect baseline data on mental health status and QOL. Lastly, mental distress and QOL were collected through a self-reported questionnaire rather than a clinical interview. There were no objective measurements for fatigue. Taken together, a further longitudinal, multi-center study with a more comprehensive assessment of mental health and QOL is warranted to evaluate the long-term impacts of COVID-19 on mental health.

In summary, using a network approach, our study demonstrated several key symptoms including uncontrollable and excessive worry, psychomotor symptoms, intrusion, daytime dysfunction, loss of energy, and mental fatigue that played a vital role in the mental distress and QOL among COVID-19 survivors. Prompt screening and targeted interventions for these symptoms might hold great promise in preventing mental distress and improving QOL in this population.

## Data availability statement

The raw data supporting the conclusions of this article will be made available by the authors, without undue reservation.

## Ethics statement

The studies involving human participants were reviewed and approved by Institutional Review Board of the Ethics Committee of Beijing University of Traditional Chinese Medicine (2020BZHYLL0111). The patients/participants provided their written informed consent to participate in this study.

## Author contributions

PP and YW: formal analysis and writing—original draft. ZL and YZ: writing—review and editing. JW, QM, and TL: conceptualization and writing–review and editing. All authors contributed to the article and approved the submitted version.

## Funding

This work was supported by the Key project of National Key Research and Development Project of the Ministry of Science and Technology “Technical Equipment for Public Security Risk Prevention and Control and Emergency Response” (2020YFC0845200). These sources had no further role in this study design, in the data collection and analysis, in the writing of the report, and in the decision to submit the paper for publication.

## Conflict of interest

The authors declare that the research was conducted in the absence of any commercial or financial relationships that could be construed as a potential conflict of interest.

The handling editor YZ declared a shared parent affiliation with the author MQ at the time of review.

## Publisher’s note

All claims expressed in this article are solely those of the authors and do not necessarily represent those of their affiliated organizations, or those of the publisher, the editors and the reviewers. Any product that may be evaluated in this article, or claim that may be made by its manufacturer, is not guaranteed or endorsed by the publisher.
