# Supplementary material for: A network analysis of the long-term quality of life and mental distress of COVID-19 survivors 1 year after hospital discharge
Source: Front Public Health. 2023 Jul 28;11:1223429. doi: 10.3389/fpubh.2023.1223429 (PMC10416228; doi:10.3389/fpubh.2023.1223429)
Supplement: Supplementary file 3 [file Presentation_1.pdf]

Supplementary materials for “**A network analysis of the long-term quality of life and mental distress of COVID-19 survivors one year after hospital discharge**”

Figure S1 Estimation of node expected difference within the network of mental distress by bootstrapped difference test Gray boxes indicate symptoms that do not significantly differ from one another. Black boxes represent symptoms that differ significantly from one another ($\alpha = 0.05$).

Figure S2 The stability of the network of mental distress The stability of central and bridge expected influence by case-dropping bootstrap.

Figure S3 The accuracy of the edges estimated in the network of mental distress The accuracy of the network edges by non-parametric bootstrapping.

Figure S4 The network of mental distress and QOL after adjusting demographic information

Figure S5 The bridge linking mental distress and QOL after adjusting demographic information

Table S1 Correlation matrix of mental distress obtained in network 1

Table S2 Correlation matrix of mental distress and quality of life obtained in network2
