# Supplementary figures and images for: A network analysis of the long-term quality of life and mental distress of COVID-19 survivors 1 year after hospital discharge
Source: Front Public Health. 2023 Jul 28;11:1223429. doi: 10.3389/fpubh.2023.1223429 (PMC10416228; doi:10.3389/fpubh.2023.1223429)

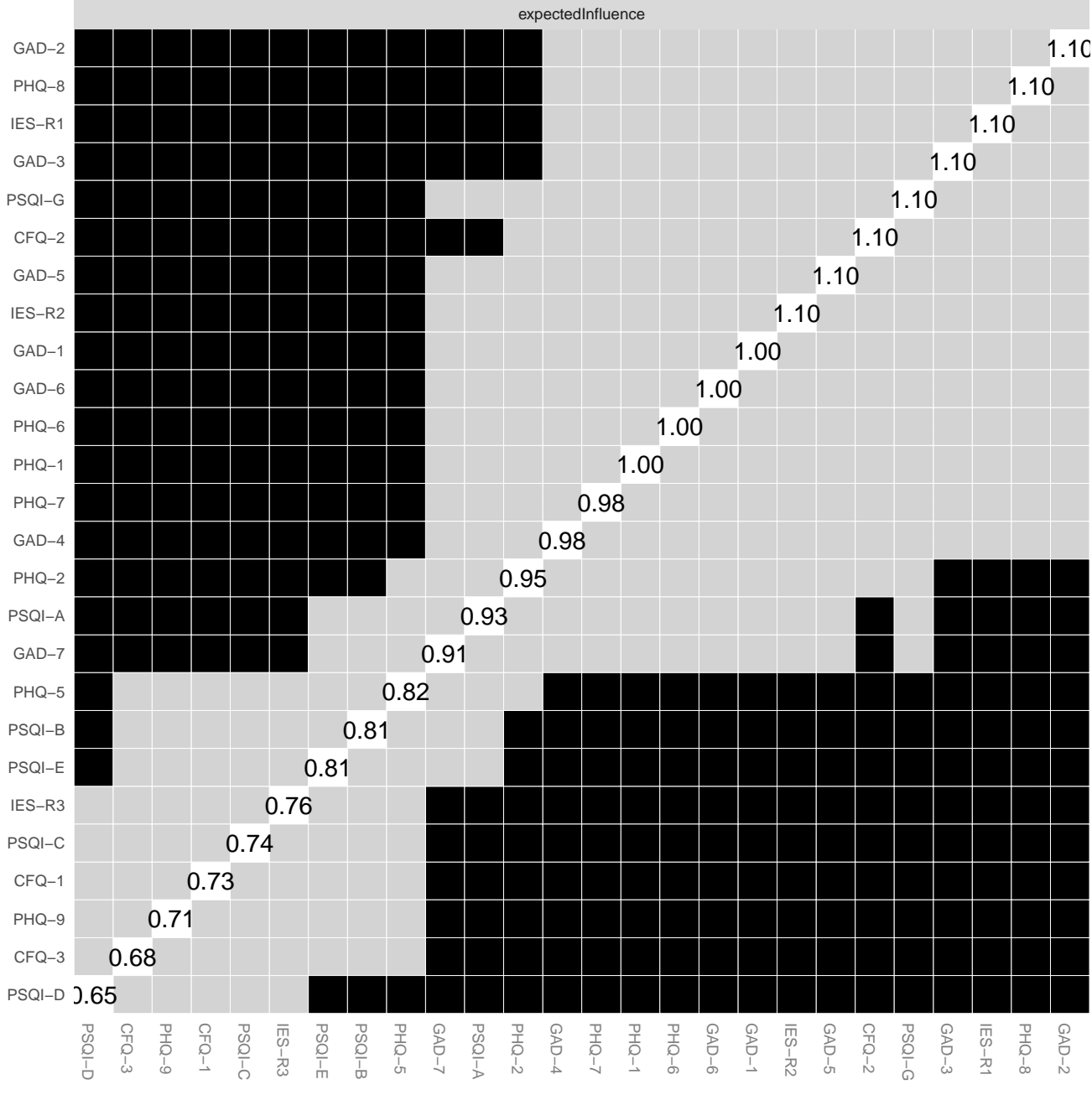

Supplement: Supplementary file 4 [file Data_Sheet_1.PDF]

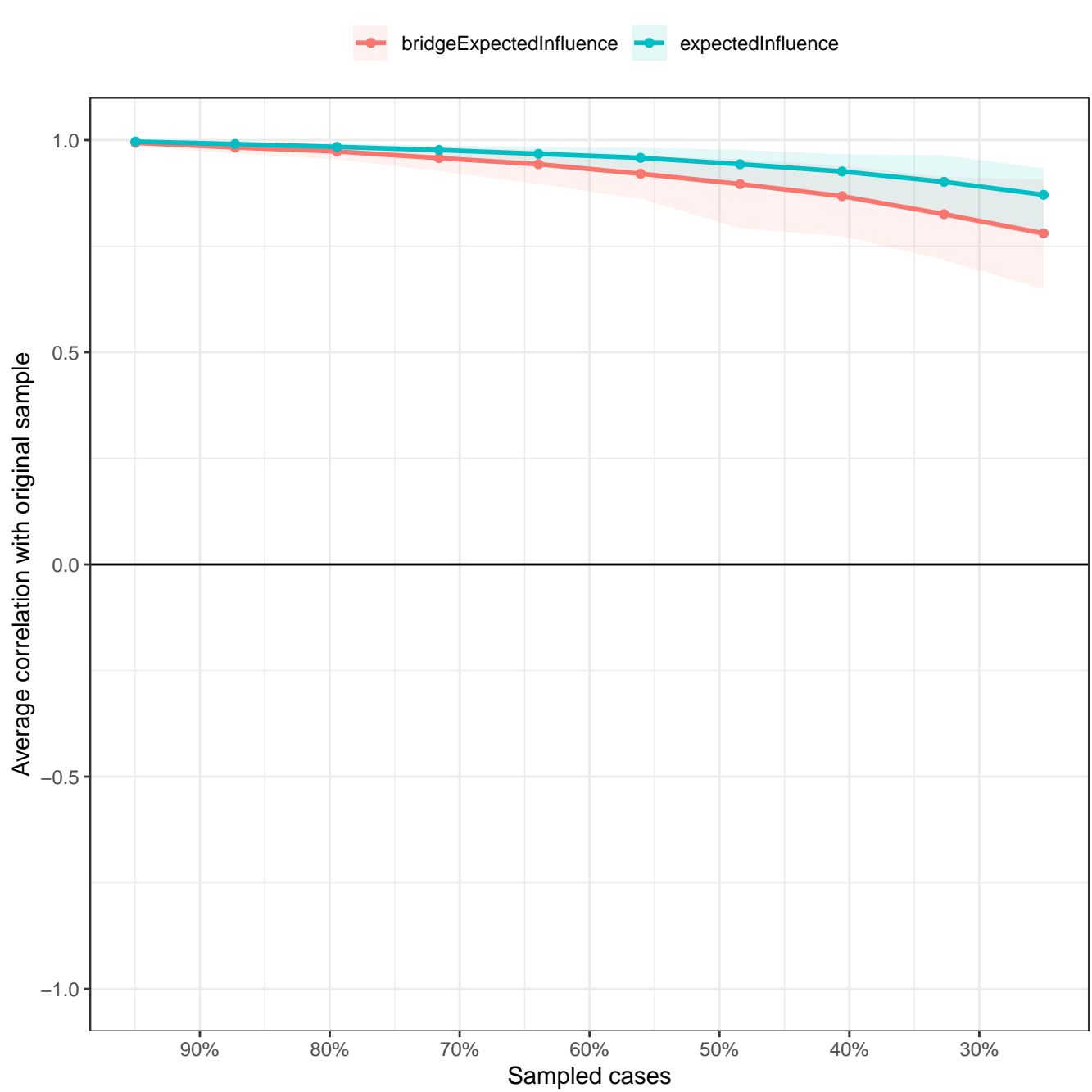

Supplement: Supplementary file 5 [file Data_Sheet_2.PDF]

● Bootstrap mean    ● Sample

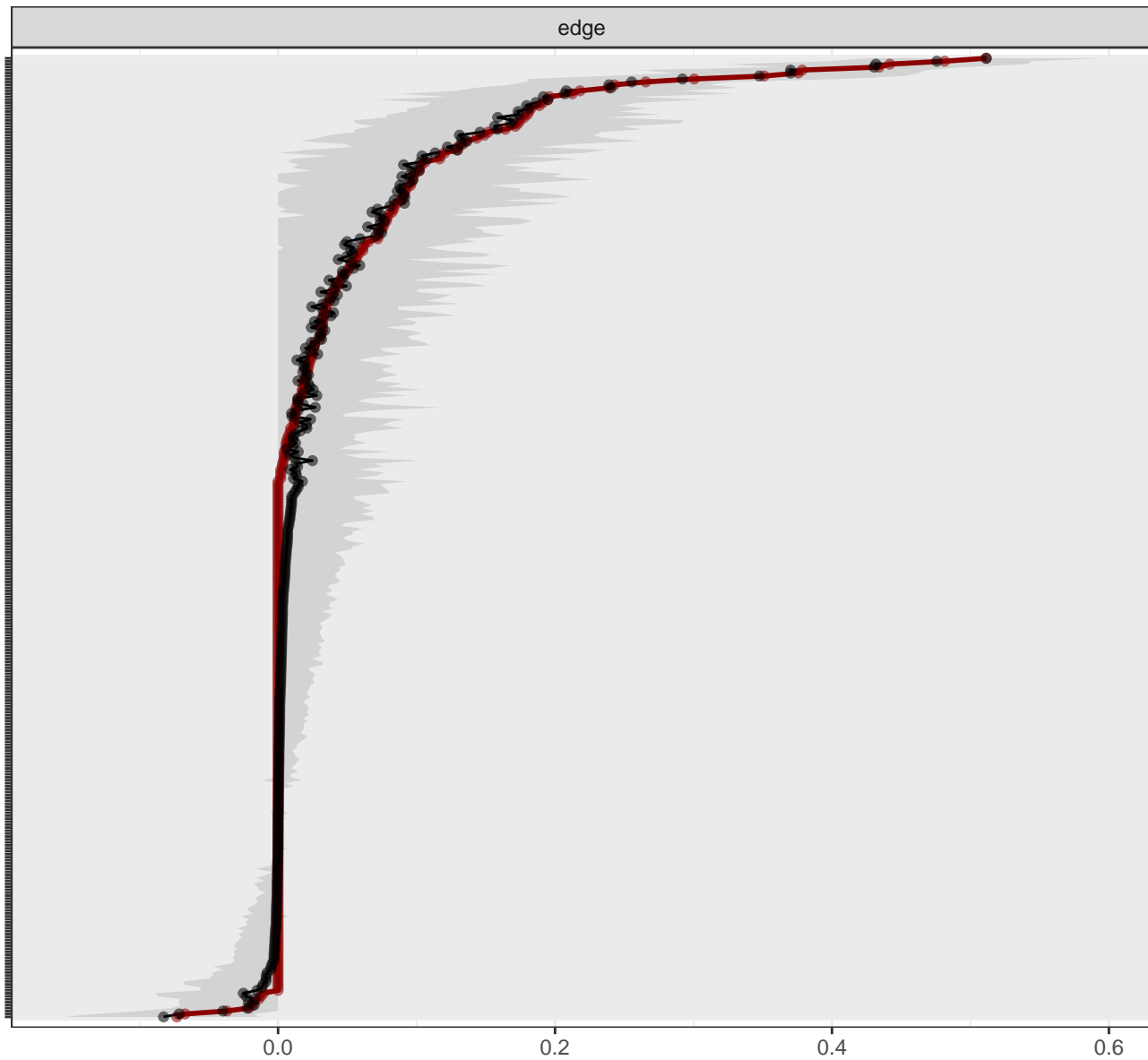

Supplement: Supplementary file 6 [file Data_Sheet_3.PDF]

Bridge Expected Influence (1-step)

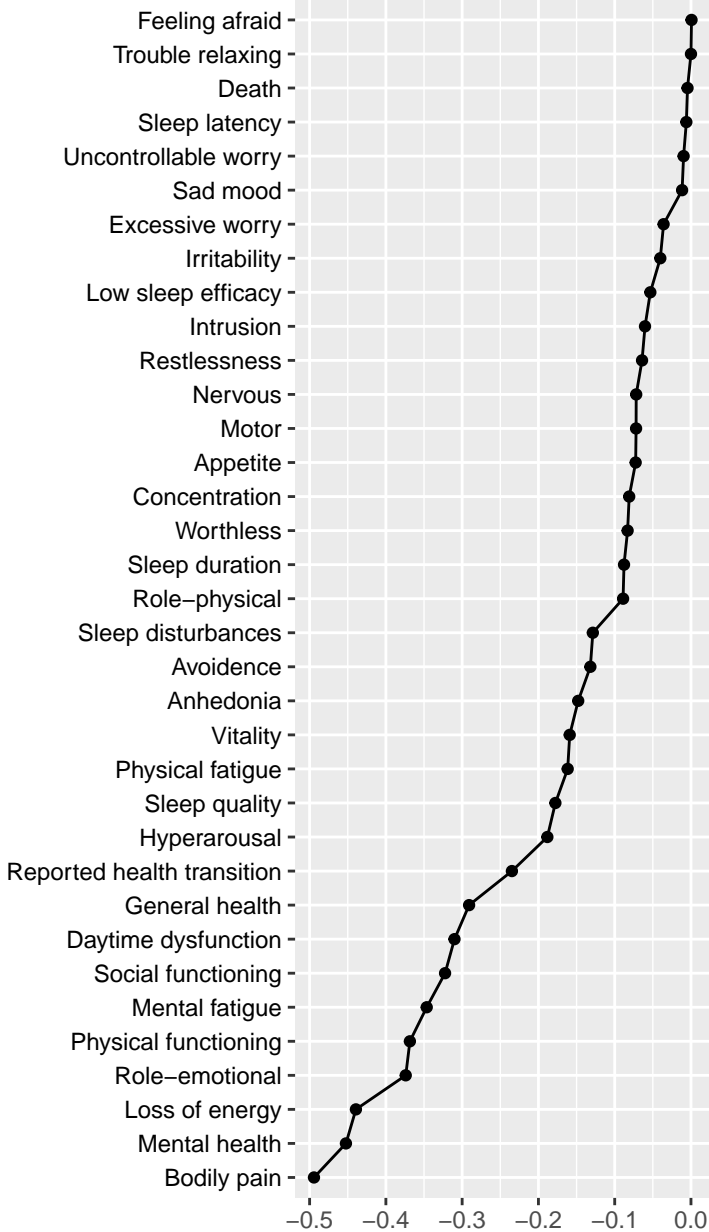

Supplement: Supplementary file 8 [file Data_Sheet_5.PDF]
