# Supplementary material for: A network analysis of the long-term quality of life and mental distress of COVID-19 survivors 1 year after hospital discharge
Source: Front Public Health. 2023 Jul 28;11:1223429. doi: 10.3389/fpubh.2023.1223429 (PMC10416228; doi:10.3389/fpubh.2023.1223429)
Supplement: Supplementary file 7 [file Data_Sheet_4.PDF]

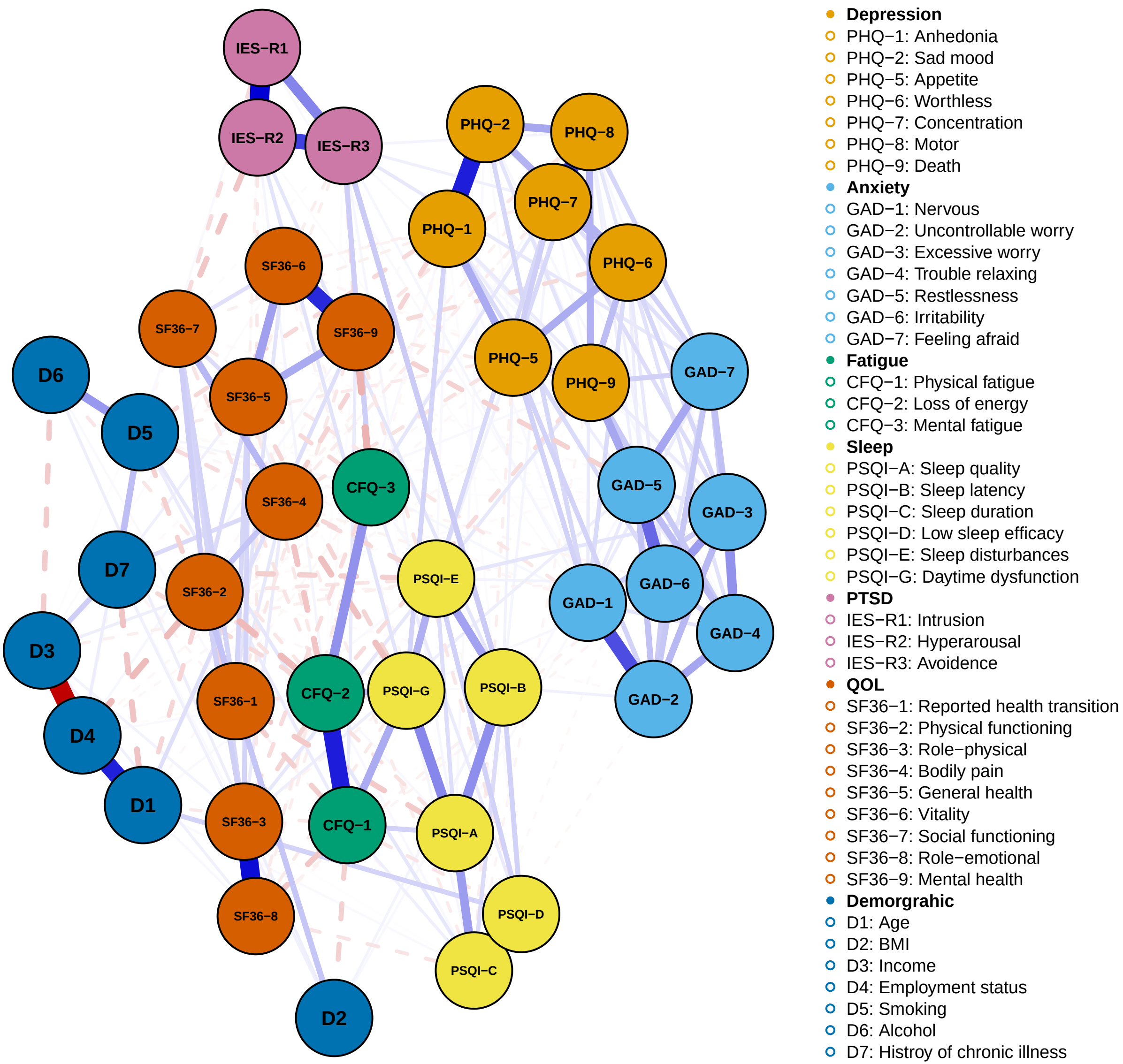
IES−R1
IES−R2
IES−R3
PHQ−2
PHQ−8
PHQ−7
PHQ−1
PHQ−6
PHQ−5
PHQ−9
SF36−6
SF36−9
SF36−7
SF36−5
SF36−4
SF36−2
SF36−1
SF36−3
SF36−8
CFQ−3
CFQ−2
CFQ−1
PSQI−E
PSQI−G
PSQI−B
PSQI−A
PSQI−D
PSQI−C
GAD−7
GAD−5
GAD−3
GAD−6
GAD−1
GAD−4
GAD−2
D6
D5
D7
D3
D4
D1
D2
Depression
PHQ−1: Anhedonia
PHQ−2: Sad mood
PHQ−5: Appetite
PHQ−6: Worthless
PHQ−7: Concentration
PHQ−8: Motor
PHQ−9: Death
Anxiety
GAD−1: Nervous
GAD−2: Uncontrollable worry
GAD−3: Excessive worry
GAD−4: Trouble relaxing
GAD−5: Restlessness
GAD−6: Irritability
GAD−7: Feeling afraid
Fatigue
CFQ−1: Physical fatigue
CFQ−2: Loss of energy
CFQ−3: Mental fatigue
Sleep
PSQI−A: Sleep quality
PSQI−B: Sleep latency
PSQI−C: Sleep duration
PSQI−D: Low sleep efficacy
PSQI−E: Sleep disturbances
PSQI−G: Daytime dysfunction
PTSD
IES−R1: Intrusion
IES−R2: Hyperarousal
IES−R3: Avoidence
QOL
SF36−1: Reported health transition
SF36−2: Physical functioning
SF36−3: Role−physical
SF36−4: Bodily pain
SF36−5: General health
SF36−6: Vitality
SF36−7: Social functioning
SF36−8: Role−emotional
SF36−9: Mental health
Demorgrahic
D1: Age
D2: BMI
D3: Income
D4: Employment status
D5: Smoking
D6: Alcohol
D7: Histroy of chronic illness
